# Characteristics of injuries during the 2006 Lebanon conflict: a three-center retrospective study of survivors, 16 years after the conflict

**DOI:** 10.3389/fpubh.2024.1382514

**Published:** 2024-05-28

**Authors:** Theresa Farhat, Hasan Nahouli, Marwan Hajjar, Zahi Abdul-Sater, Elsa Kobeissi, Marilyne Menassa, Bachar F. Chaya, Ahmad Elamine, Walaa G. El Sheikh, Hani Tamim, Shehan Hettiaratchy, Ghassan Abu-Sittah

**Affiliations:** ^1^Global Health Institute, American University of Beirut, Beirut, Lebanon; ^2^Division of Orthopedic Surgery, Department of Surgery, American University of Beirut Medical Center, Beirut, Lebanon; ^3^Division of Plastic Surgery, Department of Surgery, American University of Beirut Medical Center, Beirut, Lebanon; ^4^Clinical Research Institute, American University of Beirut Medical Center, Beirut, Lebanon; ^5^College of Medicine, Alfaisal University, Riyadh, Saudi Arabia; ^6^Centre for Blast Injury Studies, Imperial College London, London, United Kingdom; ^7^Department of Surgery and Cancer, Imperial College London, London, United Kingdom; ^8^Imperial College Healthcare NHS Trust, London, United Kingdom

**Keywords:** war, armed conflict, war injuries, disability, Lebanon

## Abstract

**Background:**

Armed conflict injury is a growing public health concern, particularly in regions like the Middle East and North Africa (MENA). The protracted conflicts and political unrest in this region have led to a substantial number of injuries. Despite this, there is still limited understanding of the specific injury patterns stemming from conflicts, such as the 2006 Lebanon conflict. This study aimed to assess the characteristics and burdens of injuries resulting from this conflict, which occurred 16 years prior to this research.

**Methods:**

This retrospective study analyzed data of individuals affected by the 2006 Lebanon conflict, across three tertiary care centers. Demographics, injuries, complications, injury management, and hospitalization expenses were extracted from medical records and analyzed using SPSS version 29.0. Categorical variables were presented as counts and proportions, and continuous variables as mean ± standard deviation (SD). Hospital comparisons utilized chi-square or Fisher’s exact tests for categorical variables, and one-way ANOVAs for continuous variables. Analysis was conducted from September to November 2023.

**Results:**

Across three hospitals, 341 patients were studied, comprising 73.6% males and 26.4% females. Among them, a notable proportion (57.3% males and 34.1% females) fell within the 18–39 age range. Children and adolescents under 18 years accounted for 15.9% of males and 25.9% of females. Blast-related injuries predominated, with 24.5% resulting from direct damage caused by explosive parts and 33.3% from blast wave forces. Extremity trauma occurred in 49.0% of patients, and head/neck trauma in 24.9%. Common injuries, including penetrating, musculoskeletal, and traumatic brain injuries affected 34.9%, 31.1, and 10.0% of patients, respectively. Wound repair, fracture treatment, and debridement were the most performed procedures on 15.5, 13.5 and 9.7% of the patients, respectively. The total cost of care was USD 692,711, largely covered by the Ministry of Public Health (95.9%).

**Conclusion:**

Conflict-related injuries significantly contribute to the global burden of disease. Therefore, there is a pressing need to improve national guidelines to prioritize life-threatening cases and potential long-term disabilities. Furthermore, enhancing electronic registry systems to collect clinical data on injured patients is essential for conducting research and better understanding the needs of conflict casualties.

## Background

Armed conflicts are major cause of death, injury, and lifelong disability worldwide, particularly in countries with relatively low preparedness for disasters, such as in the Middle East and North Africa (MENA) region (1). The MENA region’s conflicts accounted for 21.4% of global conflict deaths during the 2002–2018 period (2). Prominent conflicts in the region encompass the Iran-Iraq War (1980–1988), the Gulf War (1990–1991), the Iraq War (2003–2011), the Arab Spring uprisings (2010–2012), ongoing conflicts in Syria and Yemen, and the Israel-Lebanon conflict in 2006. Additionally, sporadic clashes and the current ongoing conflict along the Lebanon-Israel border since October 2023 contribute to the region’s tumultuous history. These events have had profound consequences, causing detrimental impacts on the injured individuals in terms of impairments and disabilities, and on the community’s socioeconomic and health status (3–6).

Lebanon, a small country in the MENA, has suffered a heavy burden of injury from intra- and inter-group conflicts, which include a protracted 15-year civil war (1975–1990) and multiple invasions and wars between Israel and Lebanon (1978, 1982, 1993, 1996, 2006, ongoing conflict since 2023) (7). The 2006 conflict in Lebanon between Israel and the armed group Hezbollah, often referred to as the 2006 Lebanon War, was one of the major conflict from a round of conflicts that ravaged the country for more than 3 decades (8). It was the result of several cross-border incidents escalating into a full-scale conflict using air, sea, and land forces and involving aerial bombings, ground operations, and rocket attacks from both sides (8). The conflict lasted 33 days and caused over 1,200 deaths in Lebanon, mostly civilians, over 4,400 injured including one-third as children, and at least 1,000,000 internally displaced persons accounting for almost 25% of the Lebanese population (9). This destruction also targeted vital points of the country’s civilian infrastructure including roads, bridges, airport, Beirut port, power and fuel stations, and hospitals (8). Around 60% of hospitals were destroyed or severely damaged, and essential health center services were disrupted due to shortages in fuel, medical supplies, and personnel (10). As a result, many injured and sick people were unable to get the treatment they needed, prompting the private sector (constituting over 90% of services) and non-governmental organizations (NGOs) to expand during the conflict to fill the gap and complement the effort of the public sector (11, 12). Since October 2023, events along the Lebanon-Israel border have led to more than 58,835 internally displaced persons, 308 reported deaths, and over 1,100 injuries, with 44% due bombardment trauma, 34% from explosions, 18% from chemical exposure, and 3% from gunfire (13, 14).

Despite their devastating impact, conflict-related injuries are under-reported and under-researched, especially in the MENA region, and the associated health needs of the conflict-affected civilians are often overlooked (15). Conducting high quality health research in these settings is often challenging due to several factors including safety, access constraints, weak local research capacity, and lack of resources and collaborations (15–18). Most current evidence-based recommendations and guidelines to improve health outcomes of injured civilians are based on the experience of Western armies and medical corps. However, there is a valuable but limited literature on injury epidemiology and civilian needs in conflict-affected countries like Afghanistan, Iraq, Libya, Gaza Strip, and Syria (5, 19–22). Research on the Syrian war from 2014 to 2017 shed light on war-related brain injuries among civilians and armed personnel within Syria, primarily attributed to gunshots, and disclosed associated impairments and mortality rates (23). Moreover, a systematic search uncovered 49 reports from 18 countries, representing 58,578 conflict-related injuries sustained by civilians and local combatants from 2001 to 2020 (24). This data is crucial to plan and organize humanitarian trauma systems capable of responding to population needs, enhancing both emergency preparedness and management within these systems (7). This study, conducted from September 2020 to October 2022, aimed to assess the long-term burden of injuries from the 2006 Lebanon conflict among civilians by analyzing injury characteristics and patterns in three tertiary care centers. Additionally, it investigated the surgical treatments provided to victims and their long-term follow-up outcomes until the date of data collection. Data related to the military aspect of casualties was not collected.

## Methods

The study design and analysis were reported in accordance with the STrengthening the Reporting of OBservational studies in Epidemiology (STROBE) guidelines (25).

### Study design

This retrospective cohort study included patients who presented with conflict-related injuries to three tertiary hospitals, during the period of the 2006 Lebanon conflict (July 12, 2006-August 15, 2006). Patients’ history of follow up visits until the date of data collection was also retrospectively examined to explore the long-term burden associated with conflict-induced injury.

### Study setting

Most casualties of the 2006 conflict were admitted to district hospitals, likely due to damaged roads and ongoing attacks. The most severe damage was observed in Tyre, Marjayoun, Nabatieh, Bint Jbeil, West Bekaa (South Lebanon), and Akkar (North). Roughly 50–70% of primary health facilities in Nabatieh and Bint Jbeil were destroyed. Most cases (57.4%, *n* = 2,549/4,400) were spread among 61 hospitals across Lebanon, with a notable concentration (67%) in hospitals in South Lebanon (12). In our study, data was collected retrospectively from three hospitals selected for retaining patient records electronically 16 years after the conflict. These hospitals were chosen strategically to represent various practices and were in primary areas that received injured patients during the war. Other hospitals either lacked accessible data due to disposal of records or declined to share due to sensitivities.

Hospital A is a non-governmental organization (NGO)-run large tertiary care center with approximately 250 beds, focused on serving marginalized and underserved populations, including refugees and those with limited financial means. It is in one of the most affected regions in the North of Lebanon during the 2006 conflict. The hospital received many casualties for primary treatment and management.

Hospital B, a private tertiary care center situated in an urban setting, is a large center for trauma care in the MENA region, with approximately 400 beds. It primarily caters to urban and affluent communities. Access to care is typically dependent on the financial capacity to afford private healthcare services, often supported by medical insurance coverage. It was considered as the main referral center during the 2006 conflict, as it received many casualties for initial management as well as victims transferred from other hospitals to receive secondary interventions and treatment.

Hospital C is a public hospital and one of the country’s main healthcare facilities with approximately 500 beds. It aimed to provide accessible care to a wider range of patients, including vulnerable populations. However, the hospital’s capacity and resources were often stretched due to high patient demand. It was one of the main centers in Beirut, the capital of Lebanon that were tasked with monitoring and managing the casualties of the 2006 conflict.

### Data collection and variables

The study included data from the electronic medical records of patients who sustained conflict-related injuries during the 2006 Lebanon conflict and presented at the selected hospitals. Data on age, sex, marital status, place of residence, mechanism of injury, location of injury, number of admissions, surgeries, injury management, associated complications, length of hospital stay, mortality, and total hospital expenses were collected. A comprehensive review of medical records for injured patients was conducted, examining entries from the onset of the conflict through October 2022. This ensured the inclusion of all follow-ups, readmissions, and any longer-term complications. However, the electronic medical files were incomplete, with variations in available data across different hospitals, resulting in the collection of only the available information.

The study data was entered into the study database and independently checked and re-verified by two researchers and then analyzed. The data collection was conducted from September 2020 to October 2022, hindered by frequent interruptions and challenges accessing hospital medical records amidst the COVID-19 situation. Hospital staff members retrieved patients’ data through a retrospective medical records review of patients admitted to their facilities and only fully anonymized data was shared with the research team responsible for data analysis.

Injuries were classified into five categories: primary (directly caused by the blast wave generated by an explosion, often affecting air-filled organs), secondary (shrapnel and other projectiles energized by the explosion causing projectile and non-projectile wounds), tertiary (displacement of the body or of its constituent parts), and quaternary (a miscellaneous collection of all other mechanisms such as burn blast-related injuries, and bullet injuries) (26).

### Statistical analysis

The data was entered in Microsoft Excel. Data management and analysis were conducted on the Statistical Package for the Social Sciences (SPSS) (IBM, New York, NY, USA) version 29.0. Categorical variables were presented using counts and proportions, while continuous variables were presented using mean with standard deviation (SD). Categorical variables were compared across hospitals using the chi-square test or Fisher’s exact test, while continuous variables were compared across hospitals using one-way ANOVAs. To compare follow-up periods between hospitals A and C, we used the independent-samples Mann–Whitney *U* test because of the small sample size for which follow-up data was available in each hospital. The data analysis was conducted from September to November 2023.

### Ethical approval

This study was approved by the American University of Beirut’s Institutional Review Board (IRB) (approval No. BIO-2018-0360). The IRB waived the requirement for informed consent from the patients since it is a retrospective study using routinely collected anonymous data. Permission to use the data was also obtained from the respective included tertiary healthcare centers.

### Patient involvement

No patients were involved in the conceptualisation or conduct of this study due to the nature of the study as a retrospective study.

## Results

### Patient demographics across hospitals

**Figure 1:**
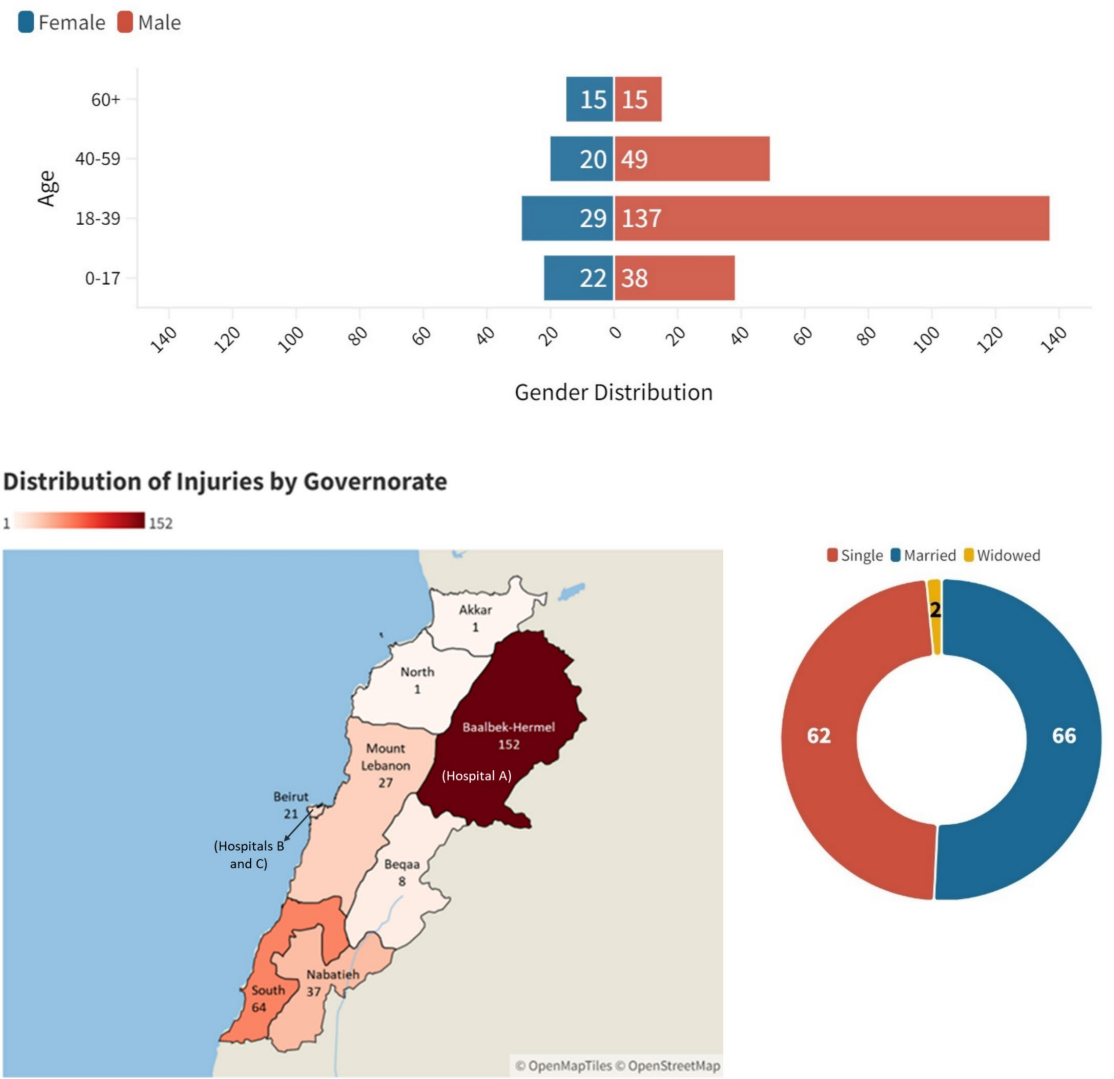
Age, gender, geographic distribution of injuries and hospitals by governorate, and marital status among conflict casualties in the three hospitals (*n* = 341).

This study included 341 participants from three hospitals (A: *n* = 194, 56.9%; B: *n* = 57, 16.7%; C: *n* = 90, 26.4%), with a male majority (73.6%, *n* = 251/341). Mean age was 32.8 ± 19.1 years. The 18–39 age group dominated (57.3% males, 34.1% females), followed by 0–17 (15.9% males, 25.9% females) (*p* < 0.001). Hospital B showed a higher percentage of single individuals at 64.3% (*n* = 9/14) compared to Hospital A (45.7%, *n* = 53/116), while Hospital C did not include the marital status in their database. Geographic origins also differed with most patients from Hospital A coming from the Baalbek-Hermel Governorate (90.2%, *n* = 148/164), while Hospital C had a substantial proportion from the South Governorate (51.1%, *n* = 46/90) and Hospital B also predominantly had patients from the South Governorate (31.6%, *n* = 18/57) (Figure 1). In-hospital mortality across all hospitals was 4.6% (*n* = 9/194) (Data not shown).

### Patterns in injury locations, types, and mechanisms among hospitalized patients

**Figure 2:**
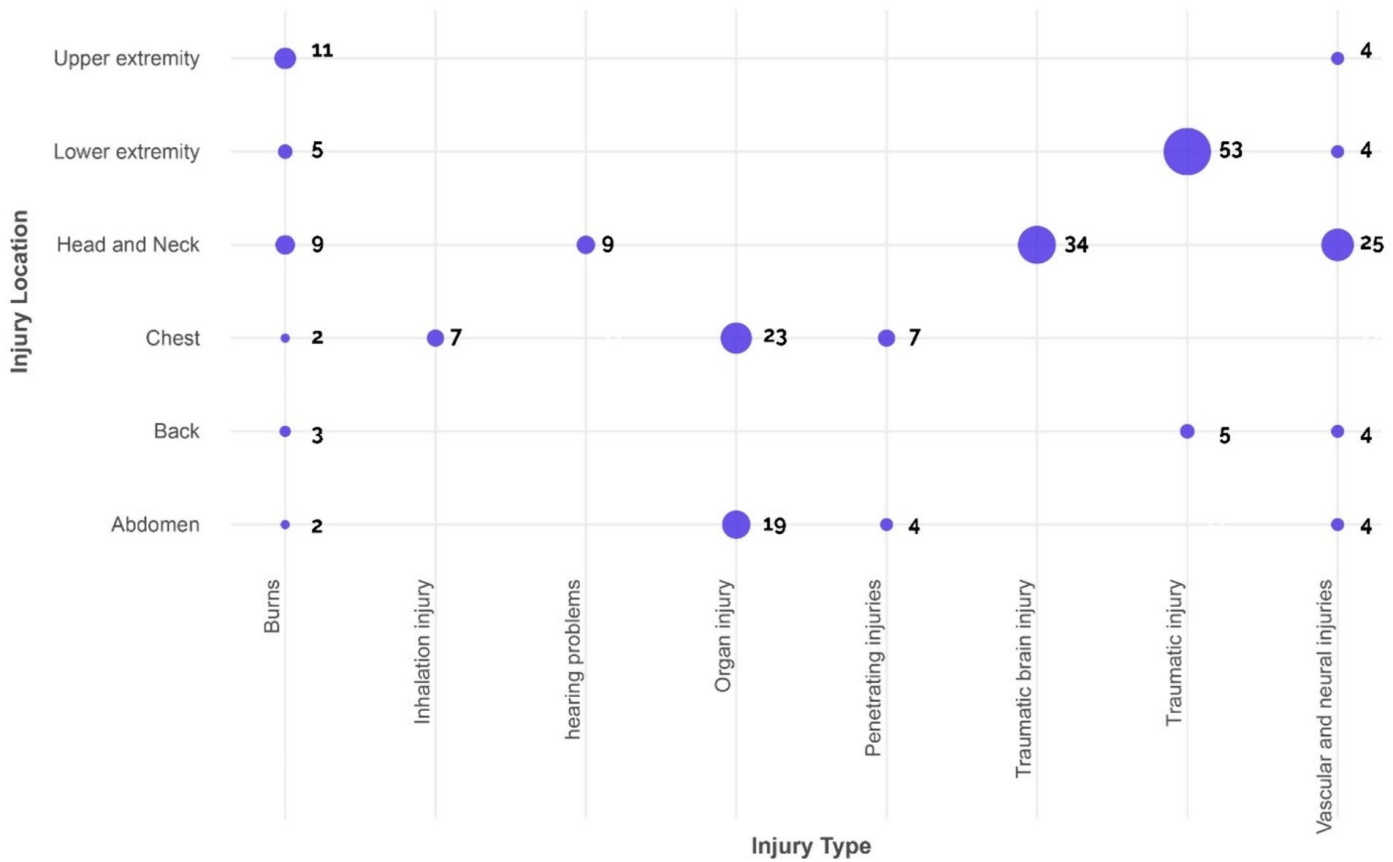
Distribution of type of injury-by-injury location in all hospitals (Unspecified locations and injury type were excluded from the figure).

This study in Hospitals A, B, and C (*n* = 341) unveiled a spectrum of injury patterns. Single injuries affected 38.7% of patients (*n* = 130/336), while 42.6% (*n* = 143/336) sustained multiple injuries across different body regions; 18.8% (*n* = 63/336) had unspecified locations. Extremity injuries dominated in 167/341 patients (49.0%), split between upper (23.5%, *n* = 80/341) and lower limbs (25.5%, *n* = 87/341) (*p* < 0.001). Head and neck injuries followed closely (24.9%, *n* = 85/341) (*p* < 0.001) (Figure 2 and Supplementary Table 1).

Injury types varied by location. Penetrating injuries (lacerations, abrasions) were most frequent in 119/341 patients (34.9%), followed by musculoskeletal injuries (fractures, dislocations, amputations) (31.1%, *n* = 106/341), and traumatic brain injuries (10.0%, *n* = 34/341). A significant portion (22.0%, *n* = 75/341) had injuries in unspecified locations (Figure 2 and Table 1).

**Table 1:** Distribution of location of injury in all patients and by hospital.

	Total number of patients				
Location of Injury – *n* (%)	All Hospitals (*N* = 341)	Hospital A (*N* = 194, 56.9%)	Hospital B (*N* = 57, 16.7%)	Hospital C (*N* = 90, 26.4%)	*p*-value
Abdomen	25 (7.3%)	9 (4.6%)	7 (12.3%)	9 (10.0%)	0.079
Back	12 (3.5%)	2 (1.0%)	6 (10.5%)	4 (4.4%)	**0.002**
Chest	41 (12.0%)	20 (10.3%)	8 (14.0%)	13 (14.4%)	0.506
Head and Neck	85 (24.9%)	33 (17.0%)	27 (47.4%)	25 (27.8%)	**<0.001**
Lower extremity	87 (25.5%)	19 (9.8%)	30 (52.6%)	38 (42.2%)	**<0.001**
Multiple spread injuries of unspecified locations	65 (19.1%)	58 (29.9%)	0 (0.0%)	7 (7.8%)	**<0.001**
Unspecified	69 (20.2%)	56 (28.9%)	0 (0.0%)	13 (14.4%)	**<0.001**
Upper extremity	80 (23.5%)	20 (10.3%)	25 (43.9%)	35 (38.9%)	**<0.001**

*P*-values in bold indicate statistical significance (<0.05).

**Figure 3:**
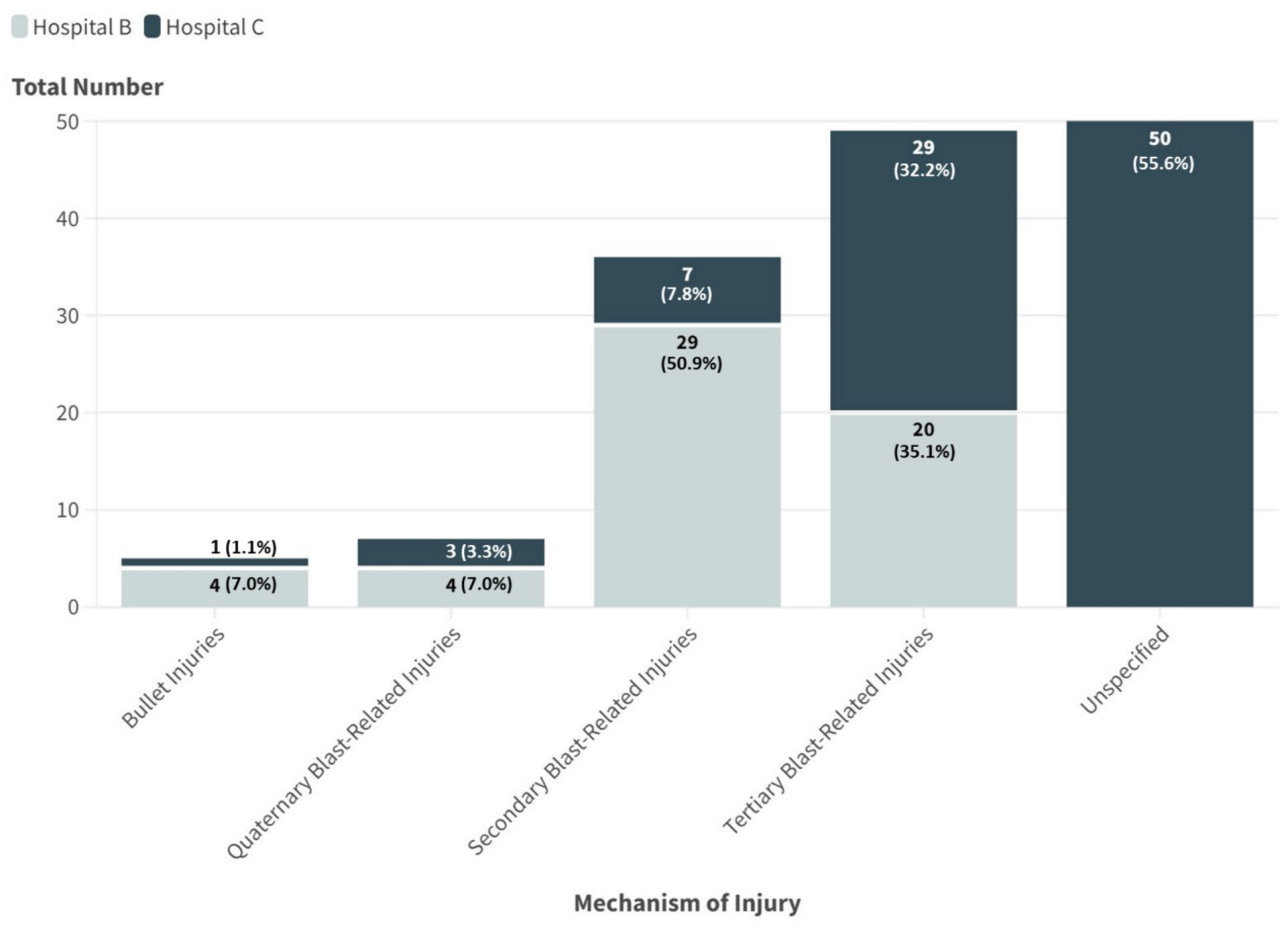
Mechanism of injury among conflict casualties across hospitals B and C (*n* = 147).

Hospital B and C displayed distinct injury mechanisms. Tertiary blast injuries, involving displacement of the body or its parts due to the explosion, were predominant (33.3%, *n* = 49/147). This was followed by secondary injuries (caused by shrapnel and other projectiles energized by the explosion, resulting in both projectile and non-projectile wounds) (24.5%, *n* = 36/147), quaternary injuries (resulting from a variety of other mechanisms such as burns or toxic inhalation) (4.8%, *n* = 7/147), and bullet injuries (3.4%, *n* = 5/147) (Figure 3). However, hospital A did not have documented injury mechanisms available for analysis.

Hospital A reported nine fatalities, including five females. Injuries varied, with 7 unspecified, 1 musculoskeletal, and 1 traumatic chest injury. The locations of injuries were unspecified in 5 cases, while 2 cases involved multiple spread injuries, 1 case involving lower extremity and another involving the chest.

### Injury management trends and disparities across hospitals

This study, encompassing 341 participants across Hospitals A, B, and C, unveiled distinct trends in injury management strategies. Open fracture care (13.5% patients, *n* = 46/341), wound repair procedures (15.5%, *n* = 53/341), and debridement (9.7%, *n* = 33/341) emerged as the mainstay interventions. Notably, complex surgical procedures like thoracic surgery and craniotomies were rarely employed in 2 instances each (0.6%), while chest tube insertions were seen in 6 patients (1.8%). Other miscellaneous approaches were employed in 2.6% (*n* = 9/341) of patients, while a concerning 15.5% (*n* = 53/341) of patients lacked documented injury management details (Table 2). Among the patients, readmission occurred for wound care, treatment of infections, cast removal, soft tissue rearrangement, as well as debridement and removal of hardware as part of injury management.

**Table 2:** Distribution of injury management; total patients and by hospital.

	Total number of patients				
Injury management – *n* (%)	All hospitals (*N* = 341)	Hospital A (*N* = 194, 56.9%)	Hospital B (*N* = 57, 16.7%)	Hospital C (*N* = 90, 26.4%)	*p*-value
Adjacent tissue rearrangement	24 (7%)	5 (2.6%)	11 (19.3%)	8 (8.9%)	**<0.001**
Amputations	2 (0.6%)	0 (0%)	2 (3.5%)	0 (0%)	**0.028**
Arthrotomy and arthrodesis	3 (0.9%)	1 (0.5%)	2 (3.5%)	0 (0%)	0.126
Chest tube insertion	6 (1.8%)	0 (0%)	5 (8.8%)	1 (1.1%)	**<0.001**
*Closed treatment of fractures	25 (7.3%)	13 (6.7%)	8 (14%)	4 (4.4%)	0.083
Craniotomy	8 (2.3%)	0 (0%)	5 (8.8%)	3 (3.3%)	**<0.001**
Debridement	33 (9.7%)	8 (4.1%)	16 (28.1%)	9 (10%)	**<0.001**
Endotracheal intubation	9 (2.6%)	9 (4.6%)	0 (0%)	0 (0%)	**0.040**
General surgery	10 (2.9%)	3 (1.5%)	5 (8.8%)	2 (2.2%)	**0.026**
Miscellaneous	9 (2.6%)	0 (0%)	9 (15.8%)	0 (0%)	**<0.001**
*Open treatment of fractures	46 (13.5%)	14 (7.2%)	16 (28.1%)	16 (17.8%)	**<0.001**
Ophthalmological procedures	7 (2.1%)	2 (1%)	4 (7%)	1 (1.1%)	**0.025**
Peripheral nerve repair	3 (0.9%)	1 (0.5%)	2 (3.5%)	0 (0%)	0.126
Removal of foreign body	15 (4.4%)	8 (4.1%)	4 (7%)	3 (3.3%)	0.581
Wound repair	53 (15.5%)	31 (16%)	18 (31.6%)	4 (4.4%)	**<0.001**
Spine surgery	2 (0.6%)	0 (0%)	2 (3.5%)	0 (0%)	**0.028**
Tendon repair	14 (4.1%)	6 (3.1%)	8 (14%)	0 (0%)	**<0.001**
Unspecified Injury management	53 (15.5%)	0 (0%)	0 (0%)	53 (58.9%)	**<0.001**
Urological reproductive	11 (3.2%)	11 (5.7%)	0 (0%)	0 (0%)	**0.011**
Vascular repair	19 (5.6%)	14 (7.2%)	4 (7%)	1 (1.1%)	0.072
Thoracic surgery	2 (0.6%)	1 (0.5%)	0 (0%)	1 (1.1%)	0.677

*The surgical treatment of fractures is done using an open approach regardless of whether the fracture was an open or closed one.

*Closed treatment of fracture is a closed reduction (without opening skin,) and/or stabilization of fracture (usually using cast).

*P*-values in bold indicate statistical significance (<0.05).

### Burden of injuries on patients and healthcare systems

This study revealed noteworthy variations in resource utilization and healthcare outcomes across the three hospitals. Length of stay (LOS) averaged 4.5 days (±5.5) but differed significantly between hospitals: Hospital A (2.2 ± 2.0 days) versus B (7.2 ± 6.8) and C (7.7 ± 7.2). Admissions were highest at Hospital C (1.9 ± 2.1) (*p* < 0.001). Hospital C had the highest follow-up rate (30.0%, *n* = 27/90), followed by Hospital B (21.1%, *n* = 12/57) and Hospital A (12.9%, *n* = 25/194) (*p* = 0.002). Mean follow-up duration was longest for Hospital C (256.88 ± 1002.07 days) compared to Hospital A (24.33 ± 26.76 days) (*p* = 0.429). Multiple injuries were most prevalent in Hospital B (61.4%, 35/57), followed by C (44.4%, 40/90) and A (35.6%, 69/194) (*p* < 0.001). Similarly, Hospital B displayed the highest mean injuries per patient (1.9 ± 1.9), compared to A (1.1 ± 0.3) and C (1.4 ± 0.7) (*p* < 0.001). Surgery rates also mirrored this trend, with Hospital B requiring the most interventions (2.5 ± 2.1) (*p* < 0.001). Approximately 40.5% of the patients (*n* = 138/341) were admitted to the emergency room (ER), while around 5.9% of the patients (*n* = 20/341) were reported to be admitted to the intensive care unit (ICU) (Table 3).

**Table 3:** Injury burden on patients and healthcare system; total patients and by hospital.

Variable	All hospitals	Hospital A	Hospital B	Hospital C	*p*-value
Length of stay (LOS) – Mean ± SD. (Missing = 1, 0.3%)	4.5 ± 5.5	2.2 ± 2	7.2 ± 6.8	7.7 ± 7.2	**<0.001**
Total number of admissions per patient – Mean ± SD.	1.4 ± 1.3	1.3 ± 0.8	1.3 ± 0.6	1.9 ± 2.1	**<0.001**
Total number of re-admissions per patient – Mean ± SD.	0.50 ± 1.3	0.27 ± 0.7	0.28 ± 0.6	1.50 ± 2.5	**<0.001**
Total number of follow-ups	64 (18.8%)	25 (12.9%)	12 (21.1%)	27 (30.0%)	**0.002**
Follow-up period – Mean ± SD.	232.83 ± 949.64	24.33 ± 26.76	-	256.88 ± 1002.07	0.429
Multiple injuries and locations – *n* (%) (Unknown = 66, 19.3%)	144 (42.6%)	69 (35.6%)	35 (61.4%)	40 (44.4%)	**<0.001**
Total number of injuries per patient – Mean ± SD.	1.3 ± 0.7	1.1 ± 0.3	1.9 ± 1.0	1.4 ± 0.7	**<0.001**
Total number of surgeries per patient – Mean ± SD. (Missing = 1, 0.3%)	0.9 ± 1.4	0.5 ± 0.8	2.5 ± 2.1	0.6 ± 0.9	**<0.001**
Emergency room (ER) admissions – *n* (%)	138 (40.5%)	51 (26.3%)	57 (100%)	30 (33.3%)	**<0.001**
Intensive-care unit (ICU) admissions – *n* (%)	20 (5.9%)	18 (9.3%)	0 (0%)	2 (2.2%)	**0.007**
Average cost per patient of initial admissions ± SD – (USD)	2759.3 ± 6525.3	699.3 ± 1255.3	9770.5 ± 10950.8	Not available	**<0.001**

*P*-values in bold indicate statistical significance (<0.05).

Healthcare costs showed significant disparities between hospitals, with considerable expense (95.9%) covered by the Ministry of Health. Initial admission costs per injury were significantly higher in Hospital B (average of 9,770.5 USD ±10,950.8) compared to Hospital A (average of 699.3 USD ±1,255.3). In Hospital A, initial hospitalization expenses totaled approximately USD 135,795. Surgical costs accounted for 17.5%, consultations and non-surgical procedures for 6.8%, and hospital services, including scans and laboratory tests, for 75.7% of the total. Hospital B reported total initial hospitalization expenses of USD 556,916, but further expense breakdown was not provided (*p* < 0.001). Notably, cost data for Hospital C was unavailable in the database (Table 3).

Complications occurred in 16.1% of patients (*n* = 55/341), including wound infections (6.2%, *n* = 21/341) and long-term issues like post-traumatic stress disorder (2.3%, *n* = 8) and disabilities (1.5% amputations [*n* = 5/341], 2.3% [*n* = 8/341] others). Interestingly, data on long-term complications were absent from Hospital A (Supplementary Table 1).

General Medicine consultations were most frequent (23.1%, *n* = 79/341), followed by Orthopedics (13.8%, *n* = 47/341), Pneumology (12.3%, *n* = 42/341), and General Surgery (11.7%, *n* = 40/341) (Supplementary Table 3).

## Discussion

The study, conducted 16 years after the 2006 Lebanon conflict, examined 341 patients across three hospitals, providing valuable insights into injury demographics, patterns, healthcare burdens, and frontline care requirements.

The study revealed a notable predominance of males, particularly among the 18–39 age group, signaling the profound impact of the conflict on young adults, affecting their employment prospects, and exacerbating economic strains. This trend mirrored observations from conflicts and wars in other parts of the MENA region, such as Iraq, Syria, and Libya, indicating consistent gender imbalances influenced by traditional norms, combat exposure, and healthcare disruptions (3, 27, 28).

Extremity and head/neck injuries emerged as the most prevalent (*p* < 0.001), echoing patterns observed in conflict zones such as Iraq, Afghanistan, and others (24, 29). This trend may serve as a crucial indicator of patients’ needs and the extent of impairment and disability. The intricate nature of multiple injuries impacting various body regions underscores the severity and complexity of the sustained traumas, a common occurrence among conflict casualties (30–32).

The most common injury mechanism was tertiary blast-related injuries, involving displacement of the body or its components, consistent with previous reports on its prevalence in modern conflicts (24, 33). Blast-related injuries predominated, potentially influenced by survival bias, where severe injuries to vital organs might lead to reduced visibility at trauma centers. The high occurrence of blast and shrapnel injuries likely stemmed from the conflict’s nature, with reports indicating that around two-thirds of victims were struck by airstrikes and missiles at homes, and nearly one-third on roads (12). Survival bias might further limit trauma center visibility for severe brain, chest, or abdominal injuries (34). Comparing these findings to recent conflicts not only highlights persistent trends in blast injuries and head/neck injuries but also underscores the evolving nature of armed conflict injuries, shaped by advancements in medical care and changing warfare tactics (24, 27).

The impact of injuries on patients and healthcare systems varied significantly across facilities, leading to differences in hospital stays, injury rates, surgeries, and incurred costs (*p* < 0.001). The study highlighted substantial variations in care quality and demographics among hospitals, notably in the cost of care. For instance, Hospital B incurred four times higher costs than Hospitals A, despite treating only one-sixth of the patients. These expense differences may stem from factors such as service costs or the types of surgeries performed, contributing to the observed disparities. The Lebanese Ministry of Public Health (MOPH) played a crucial role in covering these costs, underlining the unpredictability of the 2006 conflict and its associated hospitalization expenses. The Ministry’s decree 1/478 (12) authorized the admission of conflict casualties to all hospitals under its coverage, and it collaborated with the World Health Organization (WHO) to mobilize financial resources and donations for enhanced hospital responses during the conflict (35). However, a study from 2007 on the conflict revealed significant unmet needs for medical care, therapy, and assistive devices, particularly among older, illiterate, and uninsured individuals, despite these efforts (12). Post-injury complications, including wound infections and long-term issues such as post-traumatic stress disorder and disabilities like amputations, posed an additional burden, albeit at relatively low percentages. These occurrences are likely underreported due to limited reporting and follow-ups. Notably, Hospital A lacked data on long-term complications, possibly indicating gaps in their record-keeping or reporting or patient fragmentation across multiple facilities and loss to follow up (36). Qualitative analysis of interviews with civilians injured during the 2006 conflict revealed ongoing experiences of trauma, chronic pain, impaired mobility, psychological distress, and financial repercussions, even more than a decade later (37). This aligned with a 2007 study on the same conflict, which highlighted widespread depression, distress, and anxiety among 74% (12). The convergence of evidence emphasized the critical need for comprehensive, long-term support for victims, addressing both their physical and psychological needs. Proposed recommendations included the implementation of rehabilitation programs and the enactment of laws aimed at enhancing accessibility and employment opportunities for disabled individuals (12).

The performed surgical procedures highlighted the necessity for a proficient multi-disciplinary medical team capable of operating effectively in low-resource environments. Training initiatives should prioritize life-threatening cases and those with potential long-term disabilities. Additionally, frontline health workers require training in immediate patient care for common injuries like extremity and head trauma and should receive training in fundamental procedures such as treating fractures, debridement, and wound repair. This training equips them to deliver immediate care at primary points of contact. General Medicine emerged as the most sought-after specialty, followed by Orthopedics, Pneumology, and General Surgery, highlighting the demand for various specialties in managing such cases.

Equipping frontline health workers with essential skills and implementing standardized care approaches are crucial in conflict settings to minimize preventable deaths and long-term disabilities. However, challenges during the conflict, such as delayed access to medical care due to disrupted infrastructure and shortages of drugs, medical supplies, fuel, and power supply (35), underscore the urgent need for a robust emergency preparedness plan. This plan should include a coordinated response strategy, tailored to Lebanon’s frequent exposure to conflicts and man-made disasters. Recent studies have shown high preparedness scores across Lebanese hospitals, yet critical deficiencies persist in response strategies. These shortcomings encompass insufficient ICU and surge capacity, as well as a lack of clear command structures and coordination among hospitals and emergency medical services agencies (38). The delayed establishment of a Disaster Risk Management Unit post-2006 war, not operational until 2010, reveals governmental inefficiencies. The unit’s inadequacy during the 2020 Beirut blast underscored the importance of citizen, resident, and NGO involvement, exposing governmental shortcomings in crisis management (23). Moreover, Lebanon currently lacks a national electronic trauma registry with standardized data entry procedures. This hampers the comprehensive understanding of injury data essential for identifying gaps in healthcare systems, guiding care practices, and supporting injury research and prevention initiatives.

### Strengths and limitations

Conducting health research in conflict zones presents substantial challenges. This study, sourced from three hospitals, faces selection bias as it includes primarily surviving patients with access to care, potentially overlooking more severe injuries in heavily affected regions like Southern Lebanon (12). Additionally, relying solely on these hospitals may introduce selection bias due to the absence of data from other facilities, as other hospitals might serve diverse patient profiles. The absence of data is often attributed to destroyed or inaccessible electronic medical records— a common issue in Lebanon due to limited retention periods and lack of electronic data systems (36). Incomplete or inaccurate data within the available records hinder a comprehensive understanding of injuries, their severity, and the subsequent disabilities. The absence of injury mechanism documentation in one of the hospitals complicates improving trauma care and understanding clinical outcomes. Additionally, the lack of specialty data for treating medical personnel in one hospital adds complexity to determining necessary expertise in such situations. Moreover, data on on-scene treatments (such as tourniquet application and analgesic administration, evacuation duration, triage rule application frequency across hospitals, incidence of damage-control surgery, and severe coagulopathy presence) were not collected due to the unavailability of information. Moreover, the absence of shared data among hospitals impedes a holistic view and comprehensive patient tracking, restricting thorough analysis of treatment journeys and their outcomes. Despite data challenges, the findings reveal key insights into injury characteristics, management, and healthcare disparities in the aftermath of the 2006 Lebanon conflict.

## Conclusion

Our study delving into the aftermath of the 2006 Lebanon conflict has unveiled significant insights into injury demographics, patterns, complications, and healthcare challenges. The predominance of young males affected by extensive extremity and head/neck injuries, largely caused by tertiary blast-related mechanisms, underscores the severity of the conflict’s impact. The observed disparities in care quality and costs across facilities emphasize the urgent need for standardized care approaches and robust emergency preparedness plans. Moreover, the prevalence of missing data highlights deficiencies in reporting, emphasizing the imperative of establishing a national electronic trauma registry. Addressing these challenges is paramount to minimizing preventable deaths and disabilities and enhancing overall public health outcomes in conflict settings.

## Data availability statement

The original contributions presented in the study are included in the article/Supplementary material, further inquiries can be directed to the corresponding author.

## Ethics statement

The studies involving humans were approved by American University of Beirut Institutional Review Board. The studies were conducted in accordance with the local legislation and institutional requirements. Written informed consent for participation was not required from the participants or the participants’ legal guardians/next of kin in accordance with the national legislation and institutional requirements.

## Author contributions

TF: Data curation, Formal analysis, Investigation, Methodology, Project administration, Visualization, Writing – original draft, Writing – review & editing. HN: Formal analysis, Methodology, Writing – review & editing. MH: Formal analysis, Methodology, Writing – review & editing. ZA-S: Data curation, Methodology, Project administration, Resources, Visualization, Writing – review & editing. EK: Data curation, Methodology, Writing – review & editing. MM: Investigation, Methodology, Project administration, Resources, Writing – review & editing. BC: Data curation, Methodology, Writing – review & editing. AE: Data curation, Methodology, Writing – review & editing. WE: Formal analysis, Writing – review & editing. HT: Formal analysis, Writing – review & editing. SH: Conceptualization, Funding acquisition, Supervision, Writing – review & editing. GA-S: Conceptualization, Funding acquisition, Supervision, Writing – review & editing.
