# Supplementary material for: Characteristics of injuries during the 2006 Lebanon conflict: a three-center retrospective study of survivors, 16 years after the conflict
Source: Front Public Health. 2024 May 28;12:1382514. doi: 10.3389/fpubh.2024.1382514 (PMC11165059; doi:10.3389/fpubh.2024.1382514)
Supplement: Supplementary file 1 [file Data_Sheet_1.pdf]

Supplementary Material

Supplementary Table 1. Distribution of types of injury in all patients and by hospital.

	Total Number of Patients				
**Type of Injury** – n (%)	**All Hospitals (N = 341)**	**Hospital A (N = 194, 56.9%)**	**Hospital B (N = 57, 16.7%)**	**Hospital C (N = 90, 26.4%)**	**p-value**
Burns	21 (6.2%)	4 (2.1%)	7 (12.3%)	10 (11.1%)	**0.001**
Inhalation injury	7 (2.1%)	6 (3.1%)	1 (1.8%)	0 (0.0%)	0.303
Middle ear injury and hearing problems	8 (2.3%)	3 (1.5%)	3 (5.3%)	2 (2.2%)	0.209
Organ injury	39 (11.4%)	19 (9.8%)	9 (15.8%)	11 (12.2%)	0.180
Penetrating injuries including lacerations and abrasions	119 (34.9%)	62 (32.0%)	28 (49.1%)	29 (32.2%)	**0.047**
Tendon injury	14 (4.1%)	4 (2.1%)	8 (14.0%)	2 (2.2%)	**<0.001**
Traumatic brain injury	34 (10.0%)	14 (7.2%)	10 (17.5%)	10 (11.1%)	0.067
Musculoskeletal injury including fractures, dislocations, and amputations	106 (31.1%)	28 (14.4%)	31 (54.3%)	47 (52.2%)	**<0.001**
Unspecified	75 (22.0%)	59 (30.4%)	0 (0.0%)	16 (17.8%)	**<0.001**
Vascular and neural injuries	16 (4.7%)	5 (2.6%)	10 (17.5%)	1 (1.1%)	**<0.001**
Others	12 (3.5%)	11 (5.7%)	1 (1.8%)	0 (0.0%)	**0.026**

Supplementary Table 2. Distribution of Short and Long-Term Complications and Disabilities among hospitalized patients.

	Total Number of Patients				
**Complications** – n (%)	**All Hospitals (N = 341)**	**Hospital A (N = 194, 56.9%)**	**Hospital B (N = 57, 16.7%)**	**Hospital C (N = 90, 26.4%)**	**p-value**
**Short-term Complications**					
Cardiac arrest	7 (2.1%)	5 (2.6%)	0 (0%)	2 (2.2%)	0.758
Compartment syndrome	2 (0.6%)	0 (0%)	1 (1.8%)	1 (1.1%)	0.185
Pulmonary embolism	1 (0.3%)	0 (0%)	1 (1.8%)	0 (0%)	0.167
Unspecified early complication	1 (0.3%)	0 (0%)	0 (0%)	1 (1.1%)	0.431
Urinary tract infection	1 (0.3%)	0 (0%)	1 (1.8%)	0 (0%)	0.167
Wound infection	21 (6.2%)	0 (0%)	11 (19.3%)	10 (11.1%)	**<0.001**
**Long-term Complications**					
Acquired deformity of nose	1 (0.3%)	0 (0%)	0 (0%)	1 (1.1%)	0.431
Hemiplegia	1 (0.3%)	0 (0%)	1 (1.8%)	0 (0%)	0.167

Left foot drop	1 (0.3%)	0 (0%)	1 (1.8%)	0 (0%)	0.167
Loss of Sphincter Tone	1 (0.3%)	0 (0%)	1 (1.8%)	0 (0%)	0.167
Malunion or non union	3 (0.9%)	0 (0%)	1 (1.8%)	2 (2.2%)	0.079
Nerve injury	1 (0.3%)	0 (0%)	0 (0%)	1 (1.1%)	0.431
Post traumatic stress disorder	8 (2.3%)	0 (0%)	0 (0%)	8 (8.9%)	**<0.001**
Progressive neurologic	1 (0.3%)	0 (0%)	0 (0%)	1 (1.1%)	0.431
Retinal detachment	2 (0.6%)	0 (0%)	1 (1.8%)	1 (1.1%)	0.185
End colostomy	1 (0.3%)	0 (0%)	1 (1.8%)	0 (0%)	0.167
Lower Extremity Weakness	1 (0.3%)	0 (0%)	1 (1.8%)	0 (0%)	0.167
Subglottal stenosis	1 (0.3%)	0 (0%)	1 (1.8%)	0 (0%)	0.167
Ventriculostomy	1 (0.3%)	0 (0%)	1 (1.8%)	0 (0%)	0.167
**Disability**					
Amputation	5 (1.5%)	0 (0%)	2 (3.5%)	3 (3.3%)	**0.007**
Blindness one eye	3 (0.9%)	0 (0%)	2 (3.5%)	1 (1.1%)	**0.044**
Finger amputation	4 (1.2%)	0 (0%)	2 (3.5%)	2 (2.2%)	**0.032**
Paraplegia	1 (0.3%)	0 (0%)	1 (1.8%)	0 (0%)	0.167

Supplementary Table 3. Distribution of specialists in all and by hospital.

**Specialist** – n (%)	**All Hospitals (N = 341)**	**Hospital A (N = 194, 56.9%)**	**Hospital B (N = 57, 16.7%)**	**Hospital C (N = 90, 26.4%)**	**p-value**
Cardiology	30 (8.8%)	28 (14.4%)	Not available	2 (2.2%)	**<0.001**
General Medicine	79 (23.2%)	79 (40.7%)	Not available	0 (0%)	**<0.001**
General Surgery	40 (11.7%)	14 (7.2%)	Not available	26 (28.9%)	**<0.001**
Hematology Oncology	4 (1.2%)	0 (0%)	Not available	4 (4.4%)	**0.005**
Infectious Disease	2 (0.6%)	0 (0%)	Not available	2 (2.2%)	0.097
Neonatology Pediatrics	1 (0.3%)	0 (0%)	Not available	1 (1.1%)	0.431
Nephrology	3 (0.9%)	2 (1%)	Not available	1 (1.1%)	1.000
Neurology	32 (9.4%)	32 (16.5%)	Not available	0 (0%)	**<0.001**
Neurosurgery	8 (2.3%)	4 (2.1%)	Not available	4 (4.4%)	0.245
Obstetrics Gynecology	6 (1.8%)	5 (2.6%)	Not available	1 (1.1%)	0.619
Orthopedics	47 (13.8%)	22 (11.3%)	Not available	25 (27.8%)	**<0.001**
Otorhinolaryngology	7 (2.1%)	7 (3.6%)	Not available	0 (0%)	0.083
Pediatrics	7 (2.1%)	3 (1.5%)	Not available	4 (4.4%)	0.162
Plastic Surgery	4 (1.2%)	0 (0%)	Not available	4 (4.4%)	**0.005**
Pneumology	42 (12.3%)	40 (20.6%)	Not available	2 (2.2%)	**<0.001**
Radiology	5 (1.5%)	1 (0.5%)	Not available	4 (4.4%)	**0.030**
Urology	1 (0.3%)	0 (0%)	Not available	1 (1.1%)	0.431
Vascular Surgery	10 (2.9%)	4 (2.1%)	Not available	6 (6.7%)	**0.047**
Gastro enterology	1 (0.3%)	0 (0%)	Not available	1 (1.1%)	0.431
Internal Medicine	2 (0.6%)	2 (1%)	Not available	0 (0%)	1.000
Ophthalmology	1 (0.3%)	0 (0%)	Not available	1 (1.1%)	0.431
